# The vulvar microbiome in lichen sclerosus and high-grade intraepithelial lesions

**DOI:** 10.3389/fmicb.2023.1264768

**Published:** 2023-11-29

**Authors:** Lisa Pagan, Bertine W. Huisman, Michelle van der Wurff, Rosanne G. C. Naafs, Frank H. J. Schuren, Ingrid M. J. G. Sanders, Wiep Klaas Smits, Romy D. Zwittink, Jacobus Burggraaf, Robert Rissmann, Jurgen M. J. Piek, Jannie G. E. Henderickx, Mariëtte I. E. van Poelgeest

**Affiliations:** ^1^Centre for Human Drug Research, Leiden, Netherlands; ^2^Department of Gynaecology and Obstetrics, Leiden University Medical Center, Leiden, Netherlands; ^3^Netherlands Organisation for Applied Scientific Research (TNO), Zeist, Netherlands; ^4^Department of Medical Microbiology, Leiden University Center of Infectious Diseases (LU-CID), Leiden University Medical Center, Leiden, Netherlands; ^5^Department of Medical Microbiology, Center for Microbiome Analyses and Therapeutics, Leiden University Medical Center, Leiden, Netherlands; ^6^Leiden Amsterdam Center for Drug Research, Leiden University, Leiden, Netherlands; ^7^Department of Dermatology, Leiden University Medical Center, Leiden, Netherlands; ^8^Department of Obstetrics and Gynaecology, Catharina Cancer Institute, Eindhoven, Netherlands

**Keywords:** vulvar microbiome, vaginal microbiome, lichen sclerosus, vulvar HSIL, HPV, vulvar cancer

## Abstract

**Background:**

The role of the vulvar microbiome in the development of (pre)malignant vulvar disease is scarcely investigated. The aim of this exploratory study was to analyze vulvar microbiome composition in lichen sclerosus (LS) and vulvar high-grade squamous intraepithelial lesions (HSIL) compared to healthy controls.

**Methods:**

Women with vulvar lichen sclerosus (*n* = 10), HSIL (*n* = 5) and healthy controls (*n* = 10) were included. Swabs were collected from the vulva, vagina and anal region for microbiome characterization by metagenomic shotgun sequencing. Both lesional and non-lesional sites were examined. Biophysical assessments included trans-epidermal water loss for evaluation of the vulvar skin barrier function and vulvar and vaginal pH measurements.

**Results:**

Healthy vulvar skin resembled vaginal, anal and skin-like microbiome composition, including the genera *Prevotella*, *Lactobacillus*, *Gardnerella, Staphylococcus*, *Cutibacterium*, and *Corynebacterium*. Significant differences were observed in diversity between vulvar skin of healthy controls and LS patients. Compared to the healthy vulvar skin, vulvar microbiome composition of both LS and vulvar HSIL patients was characterized by significantly higher proportions of, respectively, *Papillomaviridae* (*p* = 0.045) and *Alphapapillomavirus* (*p* = 0.002). In contrast, the *Prevotella* genus (*p* = 0.031) and *Bacteroidales* orders (*p* = 0.038) were significantly less abundant in LS, as was the *Actinobacteria* class (*p* = 0.040) in vulvar HSIL. While bacteria and viruses were most abundant, fungal and archaeal taxa were scarcely observed. Trans-epidermal water loss was higher in vulvar HSIL compared to healthy vulvar skin (*p* = 0.043).

**Conclusion:**

This study is the first to examine the vulvar microbiome through metagenomic shotgun sequencing in LS and HSIL patients. Diseased vulvar skin presents a distinct signature compared to healthy vulvar skin with respect to bacterial and viral fractions of the microbiome. Key findings include the presence of papillomaviruses in LS as well as in vulvar HSIL, although LS is generally considered an HPV-independent risk factor for vulvar dysplasia. This exploratory study provides clues to the etiology of vulvar premalignancies and may act as a steppingstone for expanding the knowledge on potential drivers of disease progression.

## Introduction

Vulvar squamous cell carcinoma (VSCC) occurs in 1–2 per 100,000 women and has an increasing incidence with age (Hacker et al., 2012). VSCC is preceded by human papillomavirus (HPV)-related or HPV-independent precursor lesions (van de Nieuwenhof et al., 2009; de Sanjosé et al., 2013; Nitecki and Feltmate, 2018). Predominantly HPV type 16 and 18 can cause vulvar high-grade squamous intraepithelial lesions (HSIL), a premalignant condition responsible for approximately 20% of VSCC (de Sanjosé et al., 2013). These high-risk HPV (hrHPV) types are also notorious drivers of cervical dysplasia and carcinoma (Cubie, 2013). Vulvar lichen sclerosus (LS) is a chronic inflammatory condition that may promote development of differentiated vulvar intraepithelial neoplasia (dVIN), a premalignancy preceding the remaining 80% of all VSCC (Thuijs et al., 2021; Voss et al., 2021). dVIN has low disease incidence and poses a diagnostic challenge for both clinicians and pathologists. The central symptom in LS is pruritus, while scarring and anatomical changes of the labia minora and clitoral phimosis occur in severe cases. The etiology of LS remains debated, with indications of a genetic or autoimmune root cause (van de Nieuwenhof et al., 2009; Thuijs et al., 2021). A considerable amount of taboo is associated with vulvar disease, resulting in delays in clinical recognition and treatment, adding to substantial physical, sexual and psychological morbidity (Lockhart et al., 2013; Grimm et al., 2016; Pérez-López and Vieira-Baptista, 2017; Mullen et al., 2019).

Changes in microbiome composition have been associated with several disease conditions, including cancer (Feng et al., 2015; Byrd et al., 2018). The research field started off with recognition of single malignancy-driving micro-organisms (such as HPV for cervical cancer) and has expanded to include association of microbiome composition patterns to cancer development (Picardo et al., 2019). In vaginal diseases, higher grades of HPV-driven cervical dysplasia are correlated with a non-*Lactobacillus* dominated vaginal microbiome composition (Mitra et al., 2015; Piyathilake et al., 2016; Norenhag et al., 2020). Moreover, the presence of *Sneathia* spp. and *Mycoplasma* spp. has been correlated to co-infection with hrHPV types (Lee et al., 2013; Mitra et al., 2015; Audirac-Chalifour et al., 2016; Adebamowo et al., 2017; Klein et al., 2018; Łaniewski et al., 2019). These findings can expand our understanding of cervical diseases and serve as biomarkers and potential targets for drug development to treat cervical (pre)malignant disease.

In contrast to the vaginal microbiome, the vulvar microbiome and its role in the development of (pre)malignant vulvar disease is scarcely investigated and current knowledge is inconclusive (Pagan et al., 2021). The bacterial genera and species described on the healthy vulva include the genera *Lactobacillus*, *Corynebacterium*, *Staphylococcus*, and *Prevotella*, suggesting transfer from vaginal, cutaneous and intestinal origin. Current literature lacks longitudinal, case–controlled studies and elucidation of microbiome components other than bacteria, such as viruses, fungi and archaea. Therefore, the aim of this study was to describe and compare the vulvar microbiome composition by metagenomic shotgun sequencing in patients with lichen sclerosus and vulvar HSIL compared to healthy controls. Biophysical properties of the vulvar skin were additionally explored as the microbiome composition can influence the skin barrier function (Harris-Tryon and Grice, 2022).

## Methods

### Trial design and subjects

This study was part of an observational clinical trial to identify biomarkers for premalignant vulvar disease to increase the understanding of the etiology of VSCC (Huisman et al., 2023). The trial was conducted at the Centre of Human Drug Research in Leiden (the Netherlands) from February 2021 to October 2021. The Declaration of Helsinki was the guiding principle for trial execution and the study was approved by the medical ethics committee “Medisch-Ethische Toetsingscommissie Leiden Den Haag Delft” prior to initiation. Written informed consent from all participants was obtained prior to any study-related procedure.

Criteria for inclusion were women aged between 25 and 95 years with BMI <30 kg/m^2^. Patients with vulvar HSIL were required to have ≥1 demarcated lesion(s) ≥15 mm in diameter with confirmed histologic diagnosis. Patients with lichen sclerosus were considered eligible in case of a clinical and/or histological diagnosis of LS. Exclusion criteria were significant concomitant diseases, pregnancy, immunodeficiency, sexually transmitted disease, AIDS or hepatitis. Furthermore, individuals with other dermatological conditions in the genital area were excluded.

Lifestyle restrictions were incorporated to standardize vulvar conditions for microbiome sampling. A ≥ 28-day wash-out applied for systemic immunomodulatory medication and antibiotic use (topical or systemic). Wash-out for topical medication on the vulvar area was ≥14 days. Participants were instructed to refrain from sexual intercourse, vigorous exercise, applying vulvar products and shaving, waxing or other depilatory treatments at least 24 h before every visit. Additionally, they were instructed not to wash the vulvar area from midnight onwards on every visit.

In total, 10 healthy volunteers, 5 patients with HSIL and 10 patients with LS were enrolled in this observational study. Anal, vaginal and vulvar samples were obtained at a single time point for vulvar HSIL and LS patients, as well as healthy volunteers.

### Microbiome characterization

#### Sample collection

The vulvar microbiome was characterized in LS and HSIL patients as well as in the healthy volunteers. Microbiome samples were obtained using a 1 mL DNA/RNA Shield™ Collection Tube with Swab (Zymo Research, Freiburg, Germany). The pre-wetted swab with saline was rubbed along the vulvar skin for 30 s while slowly rotating the swab. Sampling locations on the vulva (i.e., labia minora, labia majora or perineum) depended on the location of the vulvar lesions (Supplementary Table S1). Healthy-appearing vulvar skin contralateral to the HSIL lesion was selected as non-lesional HSIL reference site where possible. Of note for LS, the non-hirsute vulvar skin should be considered affected, even if the skin appears healthy. Therefore, non-lesional LS sites were selected on the distal side of the labia majora toward the groin. Reference samples of the vaginal microbiome were obtained by introducing a dry swab mid-vaginally and once rotating 360 degrees along the vaginal wall, without touching the vulvar area upon introduction or removal. Another reference sample of the anal microbiome was obtained by rubbing a dry swab along the anus five times. Samples were stored in DNA/RNA shield at −80°C until DNA extraction was performed.

#### DNA extraction

DNA of vulvar, vaginal and anal swabs was extracted with the Quick-DNA Fecal/Soil Microbe Miniprep kit (Zymo Research; D6010). During DNA extraction, positive controls (D6300 ZymoBIOMICS Microbial Community standard, Zymo Research, United States) and negative controls (empty tubes) were included. In short, 600 μL BashingBead Buffer was added to the swabs and processed with Precellys 24 Homogenizer (Bertin Technologies) at 5,500 rpm for three rounds of 60 s each. Subsequently, samples were centrifuged (10,00 RCF; 1 min), 800 μL supernatant was transferred to a Zymo-Spin III-F Filter and centrifuged again (8,000 RCF; 1 min). Next, 1,200 μL Genomic Lysis Buffer was added to the filtrate. Of the resulting mixture, 800 μL was transferred to a Zymo-Spin IIC Column and centrifuged (10,000 RCF; 1 min). After discarding the flowthrough, 200 μL of DNA Pre-Wash Buffer was added to the Zymo-Spin IIC Column and centrifuged (10,000 RCF; 1 min). Five hundred microliter g-DNA Wash Buffer was added to the Zymo-Spin IIC Column and centrifuged (10,000 RCF; 1 min) after which 50 μL DNA Elution Buffer was added and centrifuged (10,000 RCF; 30 s). The eluted DNA was transferred over the same column and centrifuged (10,000 RCF; 30 s). Lastly, the eluted DNA was transferred to a prepared Zymo-Spin III-HRC Filter and centrifuged (16,000 RCF; 3 min). The resulting DNA was quantified with a Qubit 4 fluorometer (Invitrogen). In total, 89 out 90 clinical samples yielded measurable concentrations of DNA.

#### Metagenomic shotgun sequencing

DNA and additional positive sequencing controls were analyzed using metagenomic shotgun sequencing by GenomeScan (Leiden, the Netherlands). Upon sample entry, quality of samples was assessed by the Fragment Analyzer (Advanced Analytical Technologies) according to GenomeScan’s protocol. Given their low biomass, 73/97 (75%) samples passed entry quality control of >30 pg/mL (of which 15/25 (60%) anal, 25/25 (100%) vaginal, 29/39 (74%) vulvar samples, 0/4 (0%) negative controls and 4/4 (100%) positive controls), yet all samples passed library preparation quality control and were included for metagenomic shotgun sequencing.

Sequencing libraries were prepared using Illumina’s DNA PCR-Free Prep kit and checked on quality with the Fragment Analyzer. Libraries were sequenced with the Illumina NovaSeq6000 platform to a target depth of 3.3 million reads per sample.

### Biophysical assessments

#### Trans-epidermal water loss

Measurement of the trans-epidermal water loss (TEWL) determines the skin barrier function in a non-invasive manner (AquaFlux AF200 System, Biox, London, United Kingdom). The measurements were performed under standard environmental conditions and patients were acclimatized with removed clothing for ≥15 min before initiation of the measurements. All TEWL measurement conditions were constant during the study, with mean probe temperatures of 24.3°C and average humidity of 39.2%. A measurement was considered valid at the settling of the flux curves at a final steady level, as described previously (Niemeyer-van der Kolk et al., 2022).

#### Vulvar and vaginal pH analysis

Vulvar skin pH was determined using an electronic pH probe (Skin-pH-Meter PH905, Courage and Khazaka, Cologne, Germany). The average of three consecutive readings was recorded. The vaginal pH was measured by collecting vaginal fluid using a sterile Puritan swab rotated once mid-vaginally and subsequently applied to color-coded pH paper (Macherey-Nagel, pH 4.0–7.0), as described previously (Donders et al., 2007).

### Bioinformatic processing

#### Metagenomics pre-processing

Raw data were pre-processed with an in-house workflow.^1^ In short, the workflow removes the host genome reads and subsequently performs quality trimming of the reads. First, the host genome was removed using *bowtie2* (version 2.4.2) by mapping reads to the human reference genome (Langmead and Salzberg, 2012). The parameters passed in bowtie2 included “--very-sensitive-local” and reference genome “GRCh38.p7.”^2^ Unmapped, paired reads were filtered from the output using *samtools* (version 1.11) (Li et al., 2009). Subsequent filtered reads were processed with *fastp* (version 0.20.1) performing quality trimming, adapter removal and low-complexity filtering (Chen et al., 2018). *Fastp* parameters included trim “--cut-right, −-cut_window_size 4 --cut_mean_quality 20”; minlen “-l 50”; adapter “--detected_adapter_for_pe”; complexity “-y.” The mean total reads before processing, after filtering for human reads and after quality trimming were calculated per study group and sample type (Supplementary Table S2).

#### Microbial community profiling

Pre-processed reads were analyzed using *MetaPhlAn* (version 3.0.14) to profile the composition of the microbial communities and to predict read counts (Beghini et al., 2021). To profile the composition of the microbial communities, “--add_viruses” and “--unknown_estimation” were added besides default parameters. The outputs were merged with “merge_metaphlan_tables.py.” For the predicted read counts, parameters “-t rel_ab_w_read_stats,” “--add_viruses” and “--unknown_estimation” were included besides default parameters. Outputs were merged with an adapted version of the merge utility script. The pre-processed sequences mapped to 855 taxa (661 Bacteria, 152 Viruses, 33 Eukaryota and 9 Archaea).

#### Data analysis and availability

The resulting abundance tables were analyzed and visualized using R version 4.1.2 (Vienna, Austria) (Team R, 2014). For data analyses, 89 clinical samples were available (LS, *n* = 39; HSIL, *n* = 20; Healthy, *n* = 30). Shannon diversity and Chao1 richness were computed on rarefied data with the *phyloseq* package (version 1.38.0) at species level (McMurdie and Holmes, 2013). The *stat*_compare_*means* from the *ggpubr* package (version 0.4.0) was used to compute overall significant differences with a Kruskal-Wallis test and to compare means between swab sites with the Wilcoxon Rank Sum Test (Kassambara, 2023). The abundance table was transformed to compositional data with the *microbiome* package (version 1.16.0). Subsequently, the mean relative abundance of the 10 most abundant genera was visualized with *ggplot2* (version 3.3.6) for the bacterial and viral kingdoms on non-lesional and lesional skin of each study group (Lahti and Shetty, 2017; Wickham, 2023). For Linear discriminant analysis Effect Size (LEfSe), an object was created with the *phyloseq2lefse* function from the *phyloseqCompanion* package (version 1.1.) (Stagaman, 2023). Subsequent LEfSe analyses were performed until species level with default parameters (except LDA score > 4.0) on the Huttenhower lab Galaxy server to assess differences in relative abundance between lesional skin of LS patients and healthy vulvar skin, as well as lesional skin of vulvar HSIL patients and healthy vulvar skin (Segata et al., 2011). Aitchison distance was calculated for the Principal Coordinate Analyses (PCoA). On genus level data of the bacterial and viral kingdoms, Centered Log Ratio (CLR) transformation was performed using the *transform* function of the *microbiome* package. The *distance* function of the *phyloseq* package was used to generate a distance matrix with Euclidean distance. The *betadisper* function from the *vegan* package (version 2.6-4) was used to assess differences in variation between swab sites, while *adonis2* was used to assess differences in centroids of the swab sites and study groups with constrained permutations for each patient.^3^ Alluvial plots were generated by calculating the mean relative abundance of the 10 most abundant bacterial and viral genera in each swab site for LS and vulvar HSIL. Since each swab site amounted to a total relative abundance of 100%, mean relative abundances for each swab site were normalized by dividing the relative abundances by the number of swab sites included. Alluvial plots were visualized using *ggplot2* and *ggalluvial* (version 0.12.3) (Brunson, 2020).

#### Quality control

Positive and negative controls were included during DNA extraction, and additional positive controls were included during sequencing. Positive and negative DNA extraction controls were compared for mean total reads. Mean total reads of the positive controls (4,742,574) were significantly higher (Wilcoxon Rank Sum Test, *p* = 0.03) compared to the mean total reads of the negative controls (12,565). Although having significantly lower number of reads, the negative controls contained skin-derived bacterium *Cutibacterium acnes*.

Additionally, the taxonomic species composition of the positive controls was compared to each other and to the theoretical mock community composition. All species of the mock community could be identified in the positive controls, except *Bacillus subtilis*. Instead, *Bacillus intestinalis* was identified, which has previously been reported as expected misclassification of *Bacillus subtilis* using the MetaPhlAn taxonomy database (Wright et al., 2023). The bacterial species are present in equal ratio’s, indicating overrepresentation (>15% increase) of *Lactobacillus fermentum* and underrepresentation (>15% decrease) of *Pseudomonas aeruginosa*, *Enterococcus faecalis*, *Staphylococcus aureus*, *Listeria monocytogenes*, *Saccharomyces cerevisiae* and *Cryptococcus neoformans* (Supplementary Figure S1).

#### Data availability statement

All pre-processed metagenomics data have been deposited in the *European Nucleotide Archive* under accession number PRJEB61325.

## Results

### Cohort characteristics

In total, 25 women were included in the study. Baseline characteristics were comparable between groups (Table 1). Menopausal status and age were equally distributed. Most (24/25) participants were of Caucasian descent, with one healthy volunteer of mixed Caucasian and Latin American descent. All LS and vulvar HSIL patients had previously undergone one or multiple treatments for their vulvar condition, while healthy volunteers were naïve to any treatments of the vulvar skin.

**Table 1:** Baseline characteristics.

Characteristics	Healthy control (*N* = 10)	Vulvar high grade squamous intraepithelial lesion (*N* = 5)	Lichen sclerosus (*N* = 10)
Age in years—mean (range)	46.5 (25–73)	46.6 (32–66)	50.3 (25–72)
Pre-menopausal	5	3	5
Post-menopausal	5	2	5
Body mass index (BMI) in kg/m^2^—mean (range)	22.8 (19.6–27.6)	26.6 (21.6–30.0)	25.7 (18–30)
Ethnicity—N (%)			
White	9 (90%)	5 (100%)	10 (100%)
Other	1 (10%)^*^	0 (0%)	0 (0%)
Smoking—N (%)			
No	9 (90%)	1 (20%)	8 (80%)
Yes	1 (10%)	4 (80%)	2 (20%)
Disease duration in years—median (range)	N/A	8 (7–25)	5.5 (1–12)
Vulvar squamous cell carcinoma in medical history	0	0	1 (10%)
Fitzpatrick skin type—N (%)			
I	1 (10%)	1 (20%)	3 (30%)
II	4 (40%)	1 (20%)	4 (40%)
III	5 (50%)	3 (60%)	3 (30%)
HPV genotype biopsy—N (%)			
HPV16	0	4 (80%)	0
HPV53	0	1 (20%)^**^	1 (10%)
Negative	10 (100%)	1 (20%)	9 (90%)
Previous vulvar treatments—N (%)			
None	10 (100%)	0	0
Yes, 1 previous treatment	0	1 (20%)	7 (70%)
Yes, 2 previous treatments	0	1 (20%)	3 (30%)
Yes, 3 previous treatments	0	1 (20%)	0
Yes, 4 previous treatments	0	1 (20%)	0
Yes, 5 previous treatments		1 (20%)	
Topical treatment	0	5^***^	9^****^
Surgical	0	5	2^*****^
Coagulation	0	1	0
Laser	0	3	0
HPV vaccination (Gardasil)	0	2	0
Estriol/estradiol (vaginal)	0	0	2

^*^Mixed Latin American and Caucasian descent; ^**^One HSIL patient was both HPV 16 and 53 positive; ^***^5x imiquimod, 1x 5-FU; ^****^2x triamcinolonacetonide, 8x dermovate; ^*****^Surgical treatment encompassed vulvectomy and introitus plasty.

### Vulvar microbial skin diversity is decreased in LS compared to healthy skin

**Figure 1:**
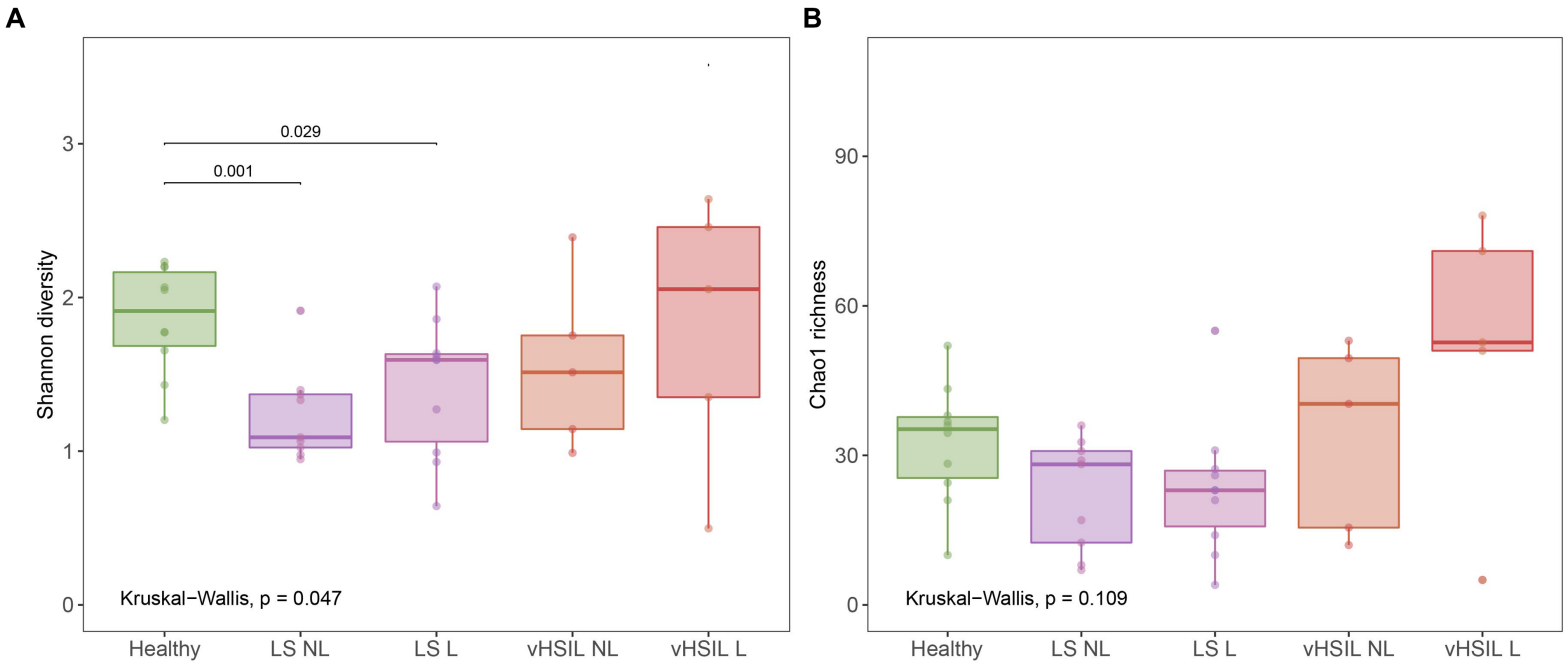
Diversity and richness of the vulvar skin microbiome. **(A)** Diversity measured by the Shannon index. **(B)** Richness measured by the Chao1 index. LS, lichen sclerosus; vHSIL, vulvar high-grade squamous intraepithelial lesions; NL, non-lesional (healthy appearing) vulvar skin; L, lesional vulvar skin. Only significant p-values are displayed.

Diversity and richness of the bacterial and viral fraction were used to assess differences in microbial ecology between vulvar skin of healthy controls and of LS and HSIL patients. Healthy vulvar skin had a higher mean diversity compared to the lesional and non-lesional skin of LS patients (*p* = 0.029 and *p* = 0.001, respectively), while other comparisons to healthy skin were not significant (Figure 1). Interestingly, within the patient groups, lesional vulvar skin showed a non-significant rise in mean diversity and richness compared to non-lesional skin.

### The bacterial fraction on diseased vulvar skin differs from healthy vulvar skin

**Figure 2:**
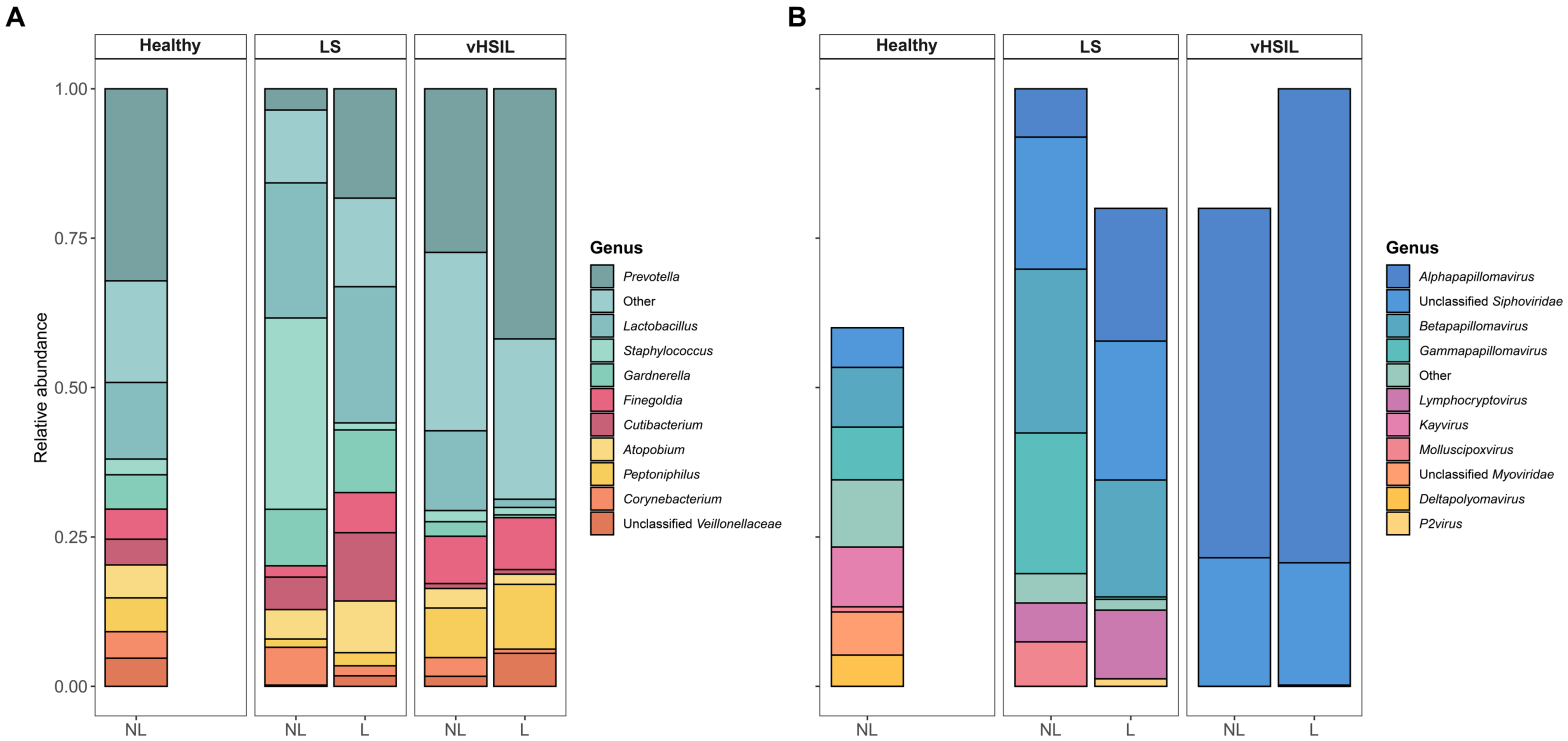
Mean relative abundance on genus level of **(A)** the bacterial fraction and **(B)** the viral fraction of the microbiome in healthy controls, LS patients and HSIL patients. For each study group, the taxonomic profiles of non-lesional and lesional vulvar skin are displayed. No viral taxa were detected in some individuals (Supplementary Figure S1), therefore the bars do not accumulate to 100% in these summary graphs. LS, lichen sclerosus; vHSIL, vulvar high-grade squamous intraepithelial lesions; NL, non-lesional (healthy appearing) vulvar skin; L, lesional vulvar skin.

To further assess differences in the microbial ecology of healthy vulvar skin and vulvar skin of LS and vulvar HSIL patients, taxonomic profiles of the bacterial and viral fraction were generated (Figure 2 and Supplementary Figures S2, S3). The bacterial and viral fraction are considered separately below.

The main bacterial genera identified in non-lesional and lesional skin of LS and vulvar HSIL patients were similar as observed in healthy vulvar skin (Figure 2A). These bacterial genera included *Prevotella*, *Lactobacillus*, *Staphylococcus*, and *Gardnerella*.

**Figure 3:**
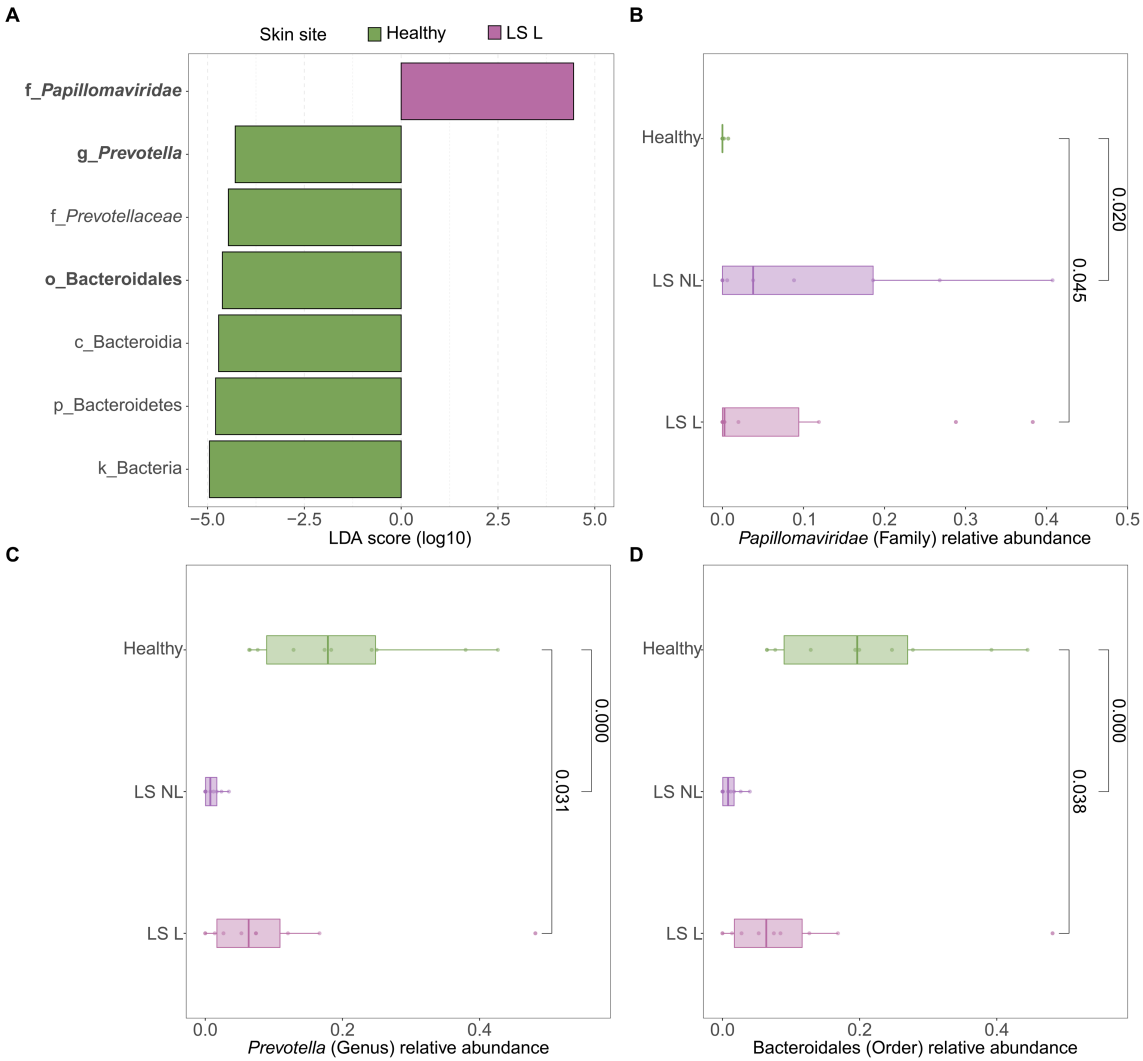
Differentially abundant features in lesional vulvar skin of lichen sclerosus patients compared to healthy controls. **(A)** LEfSe analysis histogram of LDA scores (log10) showing overrepresented taxa (pink) and underrepresented taxa (green) in lesional vulvar skin of lichen sclerosus patients. Taxa with LDA scores above 4.0 were selected and unclassified taxa were excluded for viewing purposes. Bold taxa were differential abundant features classified to the lowest taxonomic level and used for **(B–D)**. **(B–D)** Distribution of differential abundant features in individual samples per skin site with **(B)** showing overrepresented family *Papillomaviridae*, **(C)** the underrepresented genus *Prevotella* and **(D)** the underrepresented order Bacteroidales in lesional vulvar skin of lichen sclerosus patients. LS, lichen sclerosus; NL, non-lesional (healthy appearing) vulvar skin; L, lesional vulvar skin.

**Figure 4:**
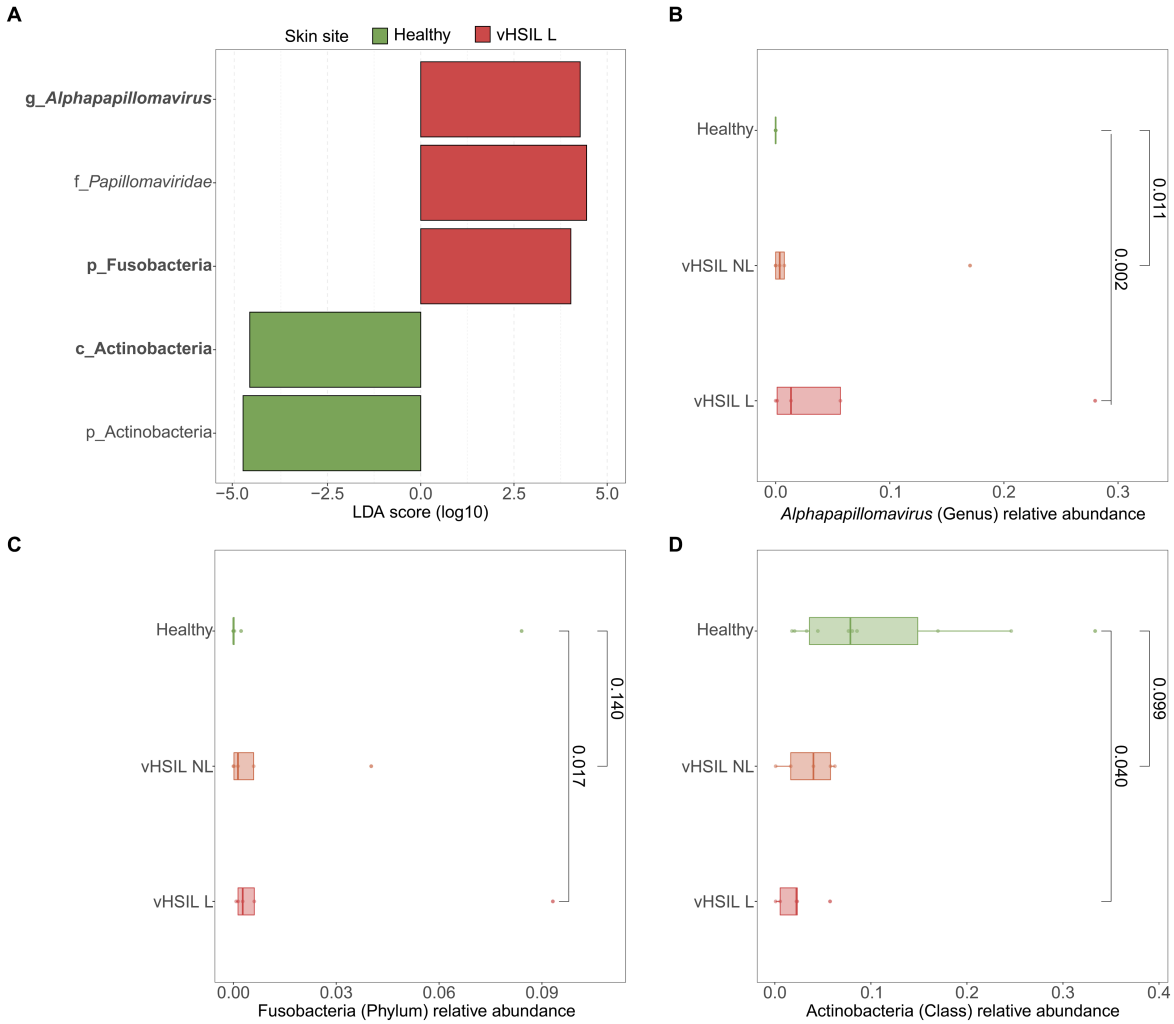
Differentially abundant features in lesional skin of vulvar HSIL patients compared to healthy controls. **(A)** LEfSe analysis histogram of LDA scores (log10) showing overrepresented taxa (red) and underrepresented taxa (green) in lesional vulvar skin of vulvar HSIL patients. Taxa with LDA scores above 4.0 were selected and unclassified taxa were excluded for viewing purposes. Bold taxa were differential abundant features classified to the lowest taxonomic level and used for **(B–D)**. **(B–D)** Distribution of differential abundant features in individual samples per skin site with **(B)** showing the overrepresented genus *Alphapapillomavirus*, **(C)** showing the overrepresented phylum Fusobacteria and **(D)** showing the underrepresented class Actinobacteria in lesional vulvar skin of vulvar HSIL patients. vHSIL, vulvar high-grade squamous intraepithelial lesions; NL, non-lesional (healthy appearing) vulvar skin; L, lesional vulvar skin.

LEfSe analyses were used to identify differentially abundant taxa between healthy vulvar skin and skin lesions in LS and vulvar HSIL. Regarding the bacterial fraction, lesional vulvar skin of LS patients was characterized by a depletion by taxa from the *Prevotella* genus and *Bacteroidales* order compared to healthy skin (Figures 3A,C,D). Meanwhile, lesional skin of HSIL patients showed an increase in the *Fusobacteria* phylum and depletion in the *Actinobacteria* class (Figures 4A,C,D).

### *Papillomaviridae* are abundant both in LS and HSIL

For the viruses, various taxa were identified on the vulva of 6/10 healthy women, although predicted read counts were relatively low compared to the bacterial reads (565 ± 892 viral reads vs. 987,765 ± 1,140,330 bacterial reads) (Supplementary Figure S4). Viruses were more often identified in LS and vulvar HSIL patients (non-lesional LS: 9/9; lesional LS: 8/10; non-lesional HSIL: 4/5; lesional HSIL: 5/5; healthy controls: 6/10). The non-lesional and lesional skin in LS and HSIL patients mainly comprised viruses within the *Papillomaviridae* family, while these were present but not dominant in healthy vulvar skin (Figure 2B). Although viruses were prevalent, the abundance of the total viral fraction on diseased skin was not significantly increased compared to healthy vulvar skin (*p* = 0.15, Wilcoxon Rank Sum Test).

LEfSe results for the viral fraction showed that *Papillomaviridae* and *Alphapapillomaviruses* were significantly more abundant in vulvar lesional skin of LS and vulvar HSIL patients compared to healthy controls, respectively (Figures 3A,B, 4A,B). More specifically, various mucosal and cutaneous HPV types were identified in LS and vulvar HSIL (Supplementary Table S3). While non-lesional and lesional vulvar skin of HSIL patients mainly contained Alphapapillomaviruses 7 and 9 species (corresponding to clinical hrHPV types 18/45 and 16/31/33, respectively), the HPV profile of LS was more diverse (Cubie, 2013). Alphapapillomaviruses 3, 6 and 13 were detected in both non-lesional and lesional vulvar LS skin. Clinically, these species correspond to several high- and low-risk HPV types, but not HPV type 16 or 18.

### *Prevotella* spp. are shared between vulvar lesional skin, the vagina and anus in lichen sclerosus and vulvar high-grade squamous intraepithelial lesions

**Figure 5:**
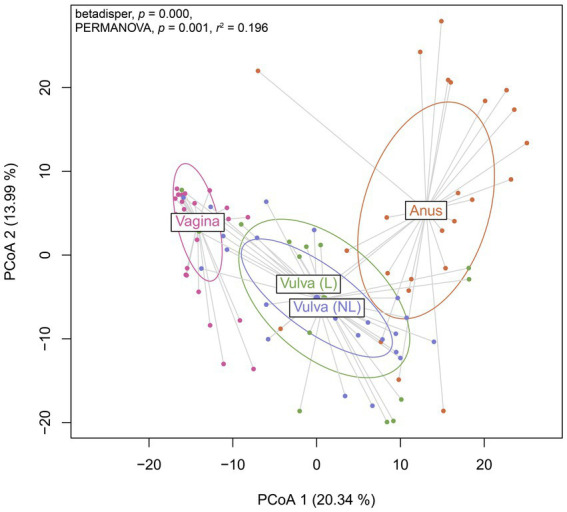
Principal Coordinates Analysis (PCoA) of Aitchison distances between bacterial and viral communities associated to the swab sites including the vagina, non-lesional (NL) and lesional (L) vulva and anus. Betadisper *p* = 0.000; PERMANOVA *p* = 0.001 and *r*^2^ = 0.196.

To further investigate the variation in the microbiome between (non-lesional and lesional) vulvar skin, vagina and anus, a Principal Coordinates Analysis was performed. The non-lesional and lesional vulvar skin showed overlap with the anus, suggesting the microbiome in vulvar skin is similar to the anal environment (Figure 5). However, PERMANOVA indicated the variation between sampling sites was significantly different (*p* = 0.001). Yet, the non-homogenous dispersion among the swab sites may have affected these PERMANOVA results.

**Figure 6:**
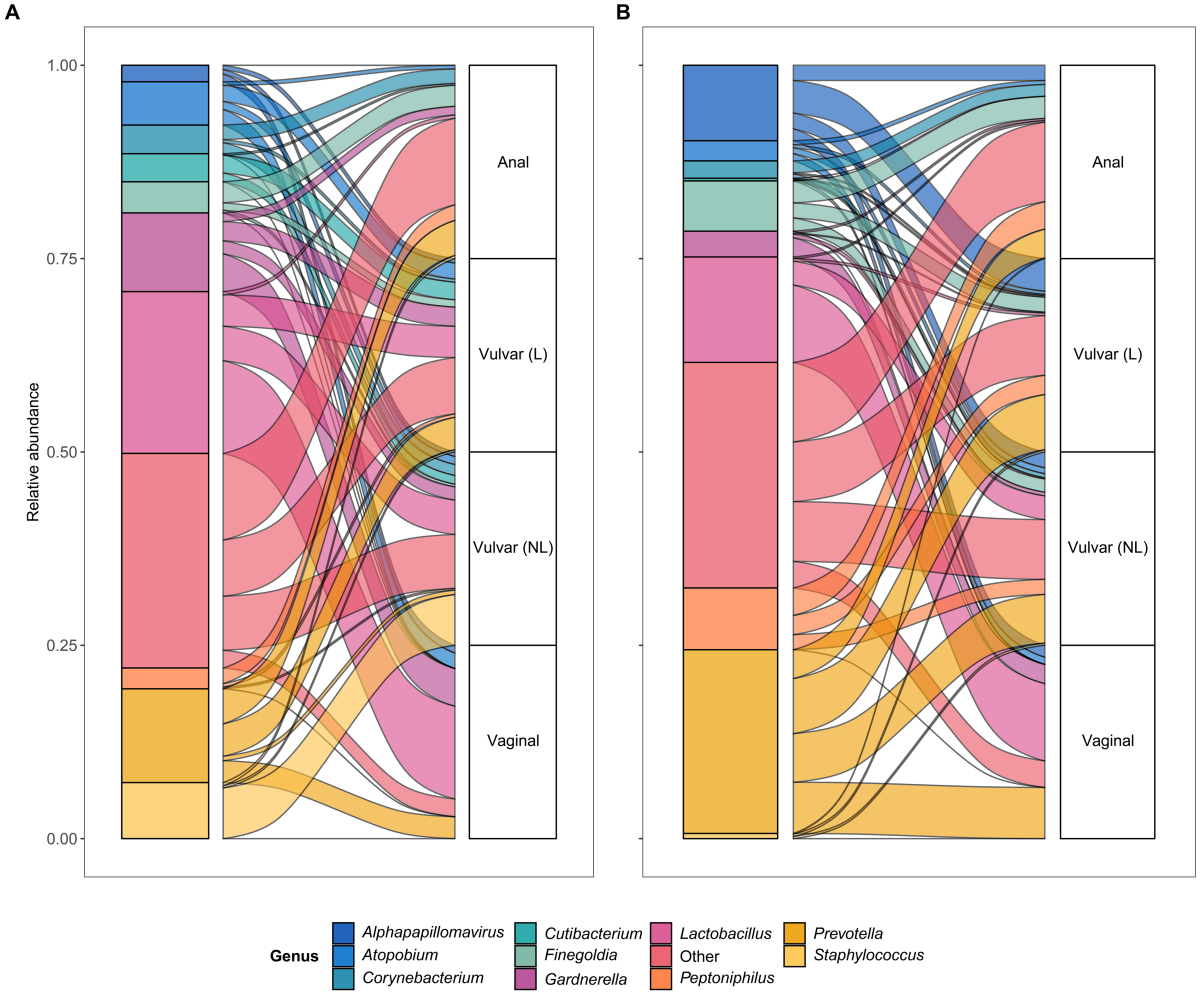
Alluvial plots showing distribution of genera over the different sampling sites in **(A)** lichen sclerosus and **(B)** vulvar high-grade squamous intraepithelial lesions (HSIL) patients.

To further assess horizontal transfer of the vaginal and anal environment to (lesional) vulvar skin and vice versa, the distribution and flow of genera was visualized (Figure 6). In both LS and HSIL patients, the *Lactobacillus* and *Gardnerella* genera were predominantly detected in the vagina and in lower relative abundances on the non-lesional and lesional skin and anus. Additionally, genera including *Campylobacter*, *Corynebacterium*, *Finegoldia* and *Gardnerella* were shared between the anus and non-lesional and lesional vulvar skin in LS and HSIL. Moreover, *Alphapapillomavirus* spp. were identified on all skin sites in vulvar HSIL patients. Interestingly, in LS patients *Prevotella* spp. was scarcely present on non-lesional vulvar skin while being detected on lesional vulvar, anal and vaginal skin sites.

### Eukaryota and Archaea are rarely detected on vulvar skin

Compared to bacterial and viral taxa, Eukaryota and Archaea were rarely identified and in low abundance (Supplementary Table S4). The most observed fungus was *Mallassezia globosa,* which was detected in low relative abundance (up to 2%) on vulvar sites of 3/10 LS patients and 1/10 healthy volunteers while being absent in the vaginal or anal milieu. *Methanobrevibacter smithii* was the most frequently found Archaea, albeit in low relative abundance (up to 1%), in anal samples of 7 participants across groups.

### The barrier function of lesional vulvar skin in vulvar HSIL is impaired

**Figure 7:**
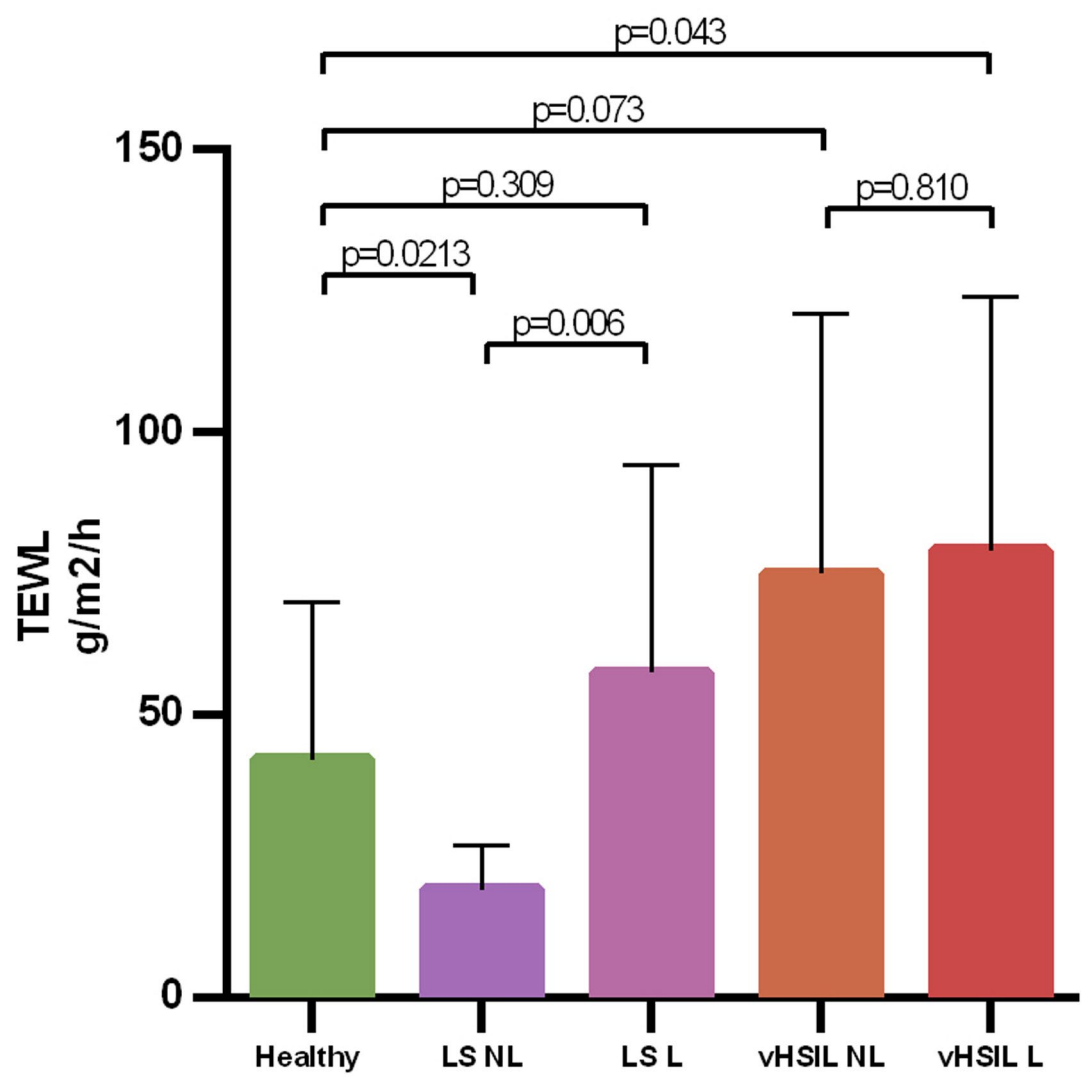
Bar graph showing the trans epidermal water loss at baseline level for all groups on de non-biopsy site. The mean and standard deviations are displayed for each group. Trans epidermal water loss in g/m^2^/h (y-axis) plotted against measurements clustered per patient group (x-axis). LS, lichen sclerosus; vHSIL, vulvar high-grade squamous intraepithelial lesions; NL, non-lesional (healthy appearing) vulvar skin; L, lesional vulvar skin.

The barrier function of the vulvar skin was assessed with TEWL. A significantly higher TEWL flux was observed between lesional vulvar HSIL [mean (SD) 79.2 g/m^2/^h (±44.9)] and healthy controls [mean (SD) 42.2 g/m^2^/h (±27.8); *p* = 0.043] (Figure 7). No significant differences in TEWL flux were observed between lesional LS [mean (SD) 57.5 g/m^2^/h (±36.8)] and HV, *p* = 0.309, nor between the non-lesional and the lesional sites of HSIL, *p* = 0.810. The lesional site of LS patients had a significant higher flux compared to the non-lesional LS site, *p* = 0.006.

The mean vulvar pH of lesional LS and HSIL was 5.72 (SD 0.45) and 6.21 (SD 0.8), respectively, with no significant changes over time (Supplementary Figure S5). Non-lesional vulvar skin and vaginal pH of patients and healthy volunteers did not display group differences or changes over time (data not shown). Menopausal status was the stratifying factor for observed differences in pH in the LS and healthy control group.

## Discussion

This exploratory study shows that the vulvar skin of patients with LS and HSIL—both non-lesional and lesional—differs from healthy vulvar skin, which was most prominent for the viral fraction. Notably, papillomaviruses were characteristic for LS, despite being considered an HPV-independent condition. In the bacterial fraction, *Prevotella* spp. were less abundant in LS than healthy vulvar skin, but shared between vulvar skin, vaginal and anal sites, indicating potential horizontal transfer between sites. This study is the first to compare the vulvar microbiome composition of LS and HSIL to healthy controls using metagenomic sequencing techniques. The distinct bacteria and viruses found in both LS and vulvar HSIL warrant further investigation and may aid in future identification of driving factors or biomarkers for vulvar (pre)malignant diseases.

The bacterial fraction of vulvar LS skin shared many taxa with healthy vulvar skin, mainly comprising *Lactobacillus*, *Prevotella*, and *Gardnerella* genera, which were also previously identified in LS and vulvodynia (Pagan et al., 2021). Specifically in vulvar LS, depletion of *Lactobacillus* and gain of *Prevotella* species has previously been described in juvenile LS (Chattopadhyay et al., 2021). The opposite was observed in this study, where taxa from the *Prevotella* genus were reduced in LS compared to healthy vulvar skin. Meanwhile within the LS group, *Prevotella* spp. were shared between anal, vaginal and lesional LS skin sites, but almost absent on non-lesional vulvar skin. Thus, the exact role of *Prevotella* in LS remains inconclusive (Chattopadhyay et al., 2021; Liu et al., 2022). Relative abundance of *Staphylococcus* spp. seemed higher in non-lesional vulvar skin, which likely reflects the more skin-like sampling location toward the groin. Based on differential abundance analysis, a significant overrepresentation of papillomaviruses and an underrepresentation of taxa from the *Prevotella* genus and the Bacteriodales order were characteristic for LS compared to healthy controls. Interestingly, LS is generally considered a HPV-independent precursor of VSCC, although concurrent HPV-infections have been previously reported (Van Der Avoort et al., 2006; Aidé et al., 2010; Hald and Blaakaer, 2018). Co-occurrence of LS and papillomaviruses may be coincidental, given the high prevalence (~10%) of both high- and low-risk genital HPV infections in the general female population (Hald and Blaakaer, 2018; Kombe Kombe et al., 2020). Alternatively, our results may point toward an etiological role of HPV in LS, in which a subclinical HPV-infection could hypothetically induce LS in genetically and/or immunologically predisposed women. Such infection could occur through disruption of the skin barrier and local immune environment, known as the Koebner phenomenon (Tasker and Wojnarowska, 2013). This could apply for both high- and low-risk HPV types as observed in LS patients of our cohort. However, no significant increase of TEWL—i.e., decrease in skin barrier function—in LS skin was observed compared to healthy controls. Another explanation for the observed co-occurrence of HPV in LS could be that skin damage or immunosuppression from corticosteroid treatment in pre-existing LS facilitates colonization with papillomaviruses. These observations in LS lead to the hypothesis that women who eventually develop VSCC may display immunological variations resulting in less effective viral clearance.

Like LS, the vulvar skin of HSIL shared bacterial genera with healthy vulvar skin. The lesional skin of vulvar HSIL was characterized by significant increases in relative abundance of the Fusobacteria phylum and *Alphapapillomavirus* genus, while the *Actinobacteria* class was significantly reduced compared to healthy controls. The presence of high-risk *Alphapapillomaviruses* corroborates with the HPV-driven etiology of vulvar HSIL. Although no data on vulvar skin sites in HSIL is available hitherto, cervical dysplasia closely relates to vulvar HSIL and has been studied extensively. In cervical dysplasia, *Sneathia*, *Mycoplasma* and *Prevotella* species have been associated with co-infection and persistence of hrHPV types (Lee et al., 2013; Mitra et al., 2015; Audirac-Chalifour et al., 2016; Adebamowo et al., 2017; Klein et al., 2018; Łaniewski et al., 2019; Ritu et al., 2019; So et al., 2020). *Prevotella* has been designated as marker genus for cervical cancer, where it may influence HPV persistence through NOD-like receptor signaling (Wu et al., 2021). These findings indicate that functional processes, driven by the microbiome, potentially contribute to persistence or progression of cervical HPV-driven diseases. No significant overrepresentation of these taxa was identified in this study to vulvar HSIL. Furthermore, microbiome composition plays an integral role in the skin barrier, interacting with its other—physical, immunological and chemical—components (Harris-Tryon and Grice, 2022). For example, TEWL can change upon topical application of *Lactobacillus* and *Corynebacterium* formulations leading to increased and reduced TEWL, respectively (2013; Park et al., 2014; Kim et al., 2021; Li et al., 2022). Our results show that TEWL was significantly increased in affected vulvar HSIL skin, indicating a disrupted skin barrier function (Akdeniz et al., 2018; Alexander et al., 2018). Whether these observed differences represent a disease-driven disturbance of skin barrier function or are due to variability in sampling location will require confirmation in an expanded population.

Besides bacteria and viruses, metagenomic sequencing allowed for the identification of Archaea and Eukaryota. Solely two Archaea were identified in low prevalence and abundance. To date, no Archaeal taxa are associated with pathogenesis and are generally considered commensals (Aminov, 2013; Wirth and Young, 2020). Eukaryotic (i.e., fungal) *Mallasezia globosa* was also identified, albeit in low relative abundance, on non-lesional vulvar sites of LS patients, but not in healthy controls or vulvar HSIL. Previously, *Mallasezia globosa* was the most identified fungal species on healthy labia majora (Bruning et al., 2020). *Candida* taxa were detected once in this study, despite the reported *C. albicans* colonization rate of 20% in the general female population (Bradford and Ravel, 2017).

The main strengths of this study are the case–control trial design including patients and healthy controls with inter-participant lesional and non-lesional control. Vaginal and anal samples allowed for intra-individual comparison and correlation of results with literature. Also, this study is the first to investigate microbiome composition in vulvar HSIL. Limitations of this exploratory study mostly pertain to the low sample size and sequencing depth. As such, future studies are needed to confirm the findings described herein. In addition, our study did not include longitudinal analyses, although we assume that the vulvar and vaginal microbiome can be particularly subject to temporal changes. Factors dictating microbiome composition fluctuations may include cycle-related changes, demographic background and lifestyle choices including sexual activity, hair removal practices and intimate hygiene routines (Ravel et al., 2011; Shiraishi et al., 2011; Gajer et al., 2012; Hickey et al., 2015; Gupta et al., 2019; Song et al., 2020; Krog et al., 2022). Lastly, the absence of certain taxa or inability to identify a proportion of the sequences may be attributed to the low biomass samples as well as the non-amplification sequencing method.

The vulvar microbiome is a growing research field, with ongoing trials in LS (NCT05671263, NCT05147129), vulvar Paget’s disease (NCT03564483) and lichen planus (NCT05330572) (ClinicalTrials.gov, 2023a,b,c,d). Future studies should strive to include a variety of vulvar diseases, such as dVIN and VSCC (HPV-positive or HPV-negative) to capture the full disease spectrum from healthy vulvar skin to VSCC. Linking microbiome findings to changes in the tumor microenvironment may be further explored, as recently reported for VSCC (Rustetska et al., 2023). Novel treatments could be developed based on microbial targets, as previously attempted for bacterial vaginosis and genitourinary symptoms (Falagas et al., 2007; Armstrong et al., 2022; Yoshikata et al., 2022). Studies to new treatment modalities, such as the current clinical trial applying the topical JAK-inhibitor ruxolitinib in LS, could consider assessing the microbiome composition as exploratory biomarker (Rissmann et al., 2020; Papp et al., 2022; ClinicalTrials.gov, 2023e). The vulvar research field to date has mainly identified presence of taxa without appraisal their involvement in biophysical or pathologic processes, which should be the focus for future studies to unravel.

## Data availability statement

The data presented in the study are deposited in the European Nucleotide Archive repository, accession number PRJEB61325.

## Ethics statement

The studies involving humans were approved by the Medisch-Ethische Toetsingscommissie Leiden Den Haag Delft. The studies were conducted in accordance with the local legislation and institutional requirements. The participants provided their written informed consent to participate in this study.

## Author contributions

LP: Conceptualization, Data curation, Formal analysis, Investigation, Methodology, Project administration, Resources, Visualization, Writing – original draft. BH: Conceptualization, Data curation, Investigation, Methodology, Project administration, Writing – review & editing. MW: Data curation, Formal analysis, Software, Visualization, Writing – review & editing, Validation. RN: Formal analysis, Investigation, Methodology, Visualization, Writing – review & editing, Data curation. FS: Conceptualization, Investigation, Methodology, Supervision, Validation, Writing – review & editing. IS: Investigation, Methodology, Project administration, Writing – review & editing. WS: Investigation, Methodology, Supervision, Writing – review & editing. RZ: Conceptualization, Investigation, Methodology, Supervision, Writing – review & editing. JB: Conceptualization, Resources, Supervision, Writing – review & editing. RR: Conceptualization, Funding acquisition, Investigation, Methodology, Resources, Supervision, Writing – review & editing. JP: Conceptualization, Investigation, Resources, Supervision, Writing – review & editing. JH: Data curation, Formal analysis, Investigation, Methodology, Software, Supervision, Validation, Visualization, Writing – original draft, Writing – review & editing. MP: Conceptualization, Funding acquisition, Investigation, Methodology, Resources, Supervision, Writing – review & editing.

## Footnotes

^1^https://git.lumc.nl/snooij/metagenomics-preprocessing

^2^https://www.ncbi.nlm.nih.gov/assembly/GCF_000001405.33/

^3^https://cran.r-project.org/web/packages/vegan/index.html
