# Erratum: The vulvar microbiome in lichen sclerosus and high-grade intraepithelial lesions

**DOI:** 10.3389/fmicb.2023.1358874

**Published:** 2024-01-09

**Authors:** Frontiers Production Office

**Affiliations:** Frontiers Media SA, Lausanne, Switzerland

**Keywords:** vulvar microbiome, vaginal microbiome, lichen sclerosus, vulvar HSIL, HPV, vulvar cancer

Due to a production error, an author's name was incorrectly spelled as “Wiep Klass Smits”. The correct spelling is “Wiep Klaas Smits”.

The publisher apologizes for this mistake. The original article has been updated.

