# Supplementary figures and images for: The vulvar microbiome in lichen sclerosus and high-grade intraepithelial lesions
Source: Front Microbiol. 2023 Nov 29;14:1264768. doi: 10.3389/fmicb.2023.1264768 (PMC10716477; doi:10.3389/fmicb.2023.1264768)

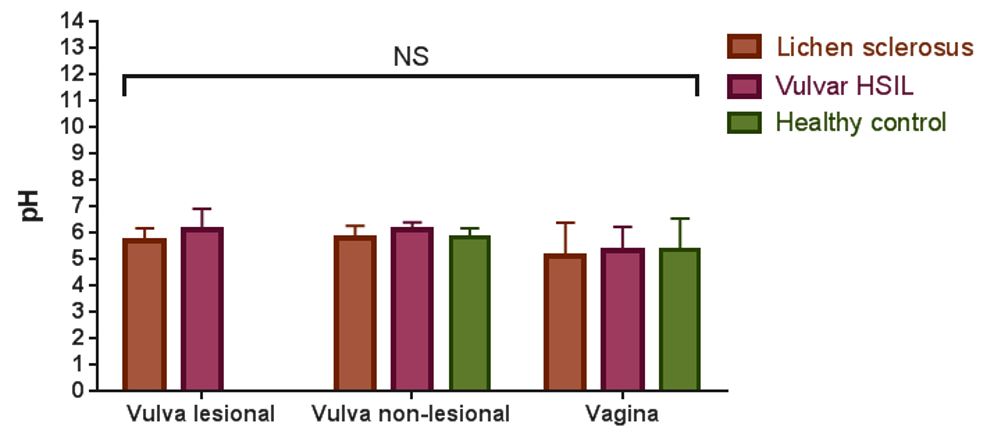

Supplement: Supplementary file 5 [file Image_5.JPEG]
