# Supplementary material for: The vulvar microbiome in lichen sclerosus and high-grade intraepithelial lesions
Source: Front Microbiol. 2023 Nov 29;14:1264768. doi: 10.3389/fmicb.2023.1264768 (PMC10716477; doi:10.3389/fmicb.2023.1264768)
Supplement: Supplementary file 6 [file Table_1.docx]

|  | | Non-lesional vulvar sample | Lesional vulvar sample |
| --- | --- | --- | --- |
| Healthy volunteers | 1 | labia majora | - |
|  | 2 | labia majora | - |
|  | 3 | labia majora | - |
|  | 4 | labia majora | - |
|  | 5 | labia majora | - |
|  | 6 | interlabial fold | - |
|  | 7 | labia majora | - |
|  | 8 | interlabial fold | - |
|  | 9 | interlabial fold | - |
|  | 10 | interlabial fold | - |
| vHSIL | 11 | interlabial fold (contralateral to lesion) | interlabial fold |
|  | 12 | labia minora (1 cm ipsilateral to lesion) | labia minora |
|  | 13 | labia minora (contralateral to lesion) | labia minora |
|  | 14 | labia majora (contralateral to lesion) | labia majora |
|  | 15 | peri-anal (1 cm ipsilateral to lesion) | peri-anal |
| Lichen sclerosus | 31 | labia majora | perineum |
|  | 32 | labia majora | labia minora |
|  | 33 | labia majora | labia minora |
|  | 34 | labia majora | labia minora |
|  | 35 | labia majora | labia minora |
|  | 36 | labia majora | labia minora |
|  | 37 | labia majora | perineum |
|  | 38 | labia majora | labia minora |
|  | 39 | labia majora | labia minora |
|  | 40 | labia majora | labia minora |

***Supplementary Table 1:*** *Vulvar sampling locations were dependent on the location of the LS and vulvar HSIL lesions. Abbreviations: LS=lichen sclerosus, vHSIL = vulvar high grade squamous intraepithelial lesion*
