# Supplementary material for: The vulvar microbiome in lichen sclerosus and high-grade intraepithelial lesions
Source: Front Microbiol. 2023 Nov 29;14:1264768. doi: 10.3389/fmicb.2023.1264768 (PMC10716477; doi:10.3389/fmicb.2023.1264768)
Supplement: Supplementary file 7 [file Table_2.docx]

***Supplementary Table 2.*** *Mean reads per study group and sample type*

| **Study group** | **Swab site** | **Mean (±SD) total Reads** | **Mean (±SD) reads after human filtering** | **Mean human filtered reads (%)** | **Mean (±SD) reads after quality trimming** |
| --- | --- | --- | --- | --- | --- |
| HV | Anal | 4,209,498 ± 776,337 | 2,913,801 ± 1,123,795 | 30.8 | 2,624,800 ± 1,050,992 |
| HV | Vulvar (NL) | 3,802,528 ± 374,790 | 1,167,062 ± 1,024,775 | 69.3 | 1,020,421 ± 951,046 |
| HV | Vaginal | 6,776,845 ± 6,049,009 | 232,400 ± 330,589 | 96.6 | 182,748 ± 292,229 |
| LS | Anal | 4,432,395 ± 1,043,317 | 1,866,656 ± 1,247,402 | 57.9 | 1,577,247 ± 1,088,727 |
| LS | Vulvar (L) | 4,449,482 ± 2,084,004 | 1,626,948 ± 2,757,321 | 63.4 | 1,446,201 ± 2,585,809 |
| LS | Vulvar (NL) | 3,720,294 ± 278,923 | 813,266 ± 604,561 | 78.1 | 642,854 ± 501,359 |
| LS | Vaginal | 7,441,991 ± 5,742,836 | 668,779 ± 1,079,685 | 91.0 | 615,314 ± 1044,238 |
| vHSIL | Anal | 3,642,566 ± 301,661 | 2,503,325 ± 569,821 | 31.3 | 2,270,303 ± 573,176 |
| vHSIL | Vulvar (L) | 11,677,054 ± 9,431,292 | 6,850,613 ± 6,663,251 | 41.3 | 6,273,799 ± 5,913,120 |
| vHSIL | Vulvar (NL) | 3,764,903 ± 320,388 | 1,488,305 ± 1,410,656 | 60.5 | 1,244,571 ± 1,191,460 |
| vHSIL | Vaginal | 5,293,525 ± 526,267 | 298,871 ± 475,807 | 94.4 | 253,227 ± 427,368 |
