# Supplementary material for: The vulvar microbiome in lichen sclerosus and high-grade intraepithelial lesions
Source: Front Microbiol. 2023 Nov 29;14:1264768. doi: 10.3389/fmicb.2023.1264768 (PMC10716477; doi:10.3389/fmicb.2023.1264768)
Supplement: Supplementary file 8 [file Table_3.docx]

***Supplementary Table 3****. Baseline characteristics.*

| **CHARACTERISICS** | **HEALTHY CONTROL**  **(N=10)** | **VULVAR HIGH GRADE SQUAMOUS INTRAEPITHELIAL LESION**  **(N=5)** | **LICHEN SCLEROSUS (N=10)** |
| --- | --- | --- | --- |
| **AGE** in years - mean (range) | 46.5 (25-73) | 46.6 (32-66) | 50.3 (25-72) |
| Pre-menopausal | 5 | 3 | 5 |
| Post-menopausal | 5 | 2 | 5 |
| Body mass index (BMI) in kg/m^2^ - mean (range) | 22.8 (19.6-27.6) | 26.6 (21.6-30.0) | 25.7 (18-30) |
| **ETHNICITY** - N (%) |  |  |  |
| White | 9 (90%) | 5 (100%) | 10 (100%) |
| Other | 1 (10%)^*^ | 0 (0%) | 0 (0%) |
| **SMOKING** - N (%) |  |  |  |
| No | 9 (90%) | 1 (20%) | 8 (80%) |
| Yes | 1 (10%) | 4 (80%) | 2 (20%) |
| **DISEASE DURATION** in years - median (range) | N/A | 8 (7-25) | 5.5 (1-12) |
| Vulvar squamous cell carcinoma in medical history | 0 | 0 | 1 (10%) |
| **FITZPATRICK skin type** - N (%) |  |  |  |
| I | 1 (10%) | 1 (20%) | 3 (30%) |
| II | 4 (40%) | 1 (20%) | 4 (40%) |
| III | 5 (50%) | 3 (60%) | 3 (30%) |
| **HPV GENOTYPE BIOPSY** - N (%) |  |  |  |
| HPV16 | 0 | 4 (80%) | 0 |
| HPV53 | 0 | 1 (20%)^**^ | 1 (10%) |
| Negative | 10 (100%) | 1 (20%) | 9 (90%) |
| **PREVIOUS VULVAR TREATMENTS** - N (%) |  |  |  |
| None | 10 (100%) | 0 | 0 |
| Yes, 1 previous treatment | 0 | 1 (20%) | 7 (70%) |
| Yes, 2 previous treatments | 0 | 1 (20%) | 3 (30%) |
| Yes, 3 previous treatments | 0 | 1 (20%) | 0 |
| Yes, 4 previous treatments | 0 | 1 (20%) | 0 |
| Yes, 5 previous treatments |  | 1 (20%) |  |
| Topical treatment | 0 | 5^***^ | 9^****^ |
| Surgical | 0 | 5 | 2^*****^ |
| Coagulation | 0 | 1 | 0 |
| Laser | 0 | 3 | 0 |
| HPV vaccination (Gardasil) | 0 | 2 | 0 |
| Estriol/estradiol (vaginal) | 0 | 0 | 2 |

^*^Mixed Latin American and Caucasian descent. ^**^One HSIL patient was both HPV 16 and 53 positive; ^***^5x imiquimod, 1x 5-FU; ^***^2x triamcinolonacetonide, 8x dermovate;
^*****^ Surgical treatment encompassed vulvectomy and introitus plasty
