# Supplementary material for: The vulvar microbiome in lichen sclerosus and high-grade intraepithelial lesions
Source: Front Microbiol. 2023 Nov 29;14:1264768. doi: 10.3389/fmicb.2023.1264768 (PMC10716477; doi:10.3389/fmicb.2023.1264768)
Supplement: Supplementary file 9 [file Table_4.docx]

**Supplementary Table 4**: Archaean and Fungal species identified across groups

|  | | | **Identified taxa** | | | | | |
| --- | --- | --- | --- | --- | --- | --- | --- | --- |
|  | | | **Fungi** | | | | **Archaea** | |
| **Patient group** | **Swab site** | **Total samples** | ***Aspergillus***  ***sydowii*** | ***Malassezia***  ***globosa*** | ***Malassezia***  ***restricta*** | ***Trichomonas***  ***vaginalis*** | ***Methano-brevibacter***  ***smithii*** | ***Methano-sphaera***  ***stadtmanae*** |
| Healthy | anal | 10 | - | - | - | - | 5 | - |
| Healthy | vulva – non-lesional | 10 | - | 1 | - | - | - | - |
| Healthy | vaginal | 10 | - | - | - | - | - | - |
| vHSIL | anal | 5 | - | - | - | - | 1 | 1 |
| vHSIL | vulva-lesional | 5 | 1 | - | - | - | - | - |
| vHSIL | vulva – non-lesional | 5 | - | - | - | - | - | - |
| vHSIL | vaginal | 5 | - | - | - | 1 | - | - |
| Lichen sclerosus | anal | 10 | - | - | - | - | - | - |
| Lichen sclerosus | vulva-lesional | 10 | - | 2 | - | - | 1 | - |
| Lichen sclerosus | vulva – non-lesional | 9 | - | 1 | 1 | - | - | - |
| Lichen sclerosus | vaginal | 10 | - | - | - | - | - | - |
