# Supplementary material for: The vulvar microbiome in lichen sclerosus and high-grade intraepithelial lesions
Source: Front Microbiol. 2023 Nov 29;14:1264768. doi: 10.3389/fmicb.2023.1264768 (PMC10716477; doi:10.3389/fmicb.2023.1264768)
Supplement: Supplementary file 10 [file Table_5.docx]

**Supplementary Table 3:** Observed Papillomaviruses detected per subgroup

| **Genus + species** | **Clinical type** | **Risk type** | **Tissue preference** | **LS** | | **vHSIL** | | **HV** |
| --- | --- | --- | --- | --- | --- | --- | --- | --- |
|  |  |  |  | **NL** | **L** | **NL** | **L** | **NL** |
| Alphapapillomavirus 10 | HPV6, HPV11 | Low-risk | Mucosal/ cutaneous |  | x |  | x |  |
| Alphapapillomavirus 13 | HPV32, HPV54 | Low-risk | Mucosal | x | x |  |  |  |
| Alphapapillomavirus 14 | HPV3, HPV10 | Low-risk | Mucosal |  |  |  |  |  |
| Alphapapillomavirus 3 | HPV3, HPV10 | Low-risk | Mucosal | x | x |  |  |  |
| Alphapapillomavirus 6 | HPV51, HPV56, HPV34 | High-risk | Mucosal | x | x |  |  |  |
| Alphapapillomavirus 7 | HPV18, HPV45 | High risk | Mucosal |  |  | x | x |  |
| Alphapapillomavirus 9 | HPV16, HPV31, HPV33, HPV52, HPV58 | High risk | Mucosal |  |  | x | x |  |
| Betapapillomavirus 1-5 | HPV 5,  HPV 8 | Mostly low risk | Cutaneous | x | x |  |  | x |
| Gammapapillomavirus 7, 8, 9, 11, 19 | HPV 4,  HPV 60 | Low-risk | Cutaneous | x | x |  |  | x |
